# Effects of Differentially Methylated CpG Sites in Enhancer and Promoter Regions on the Chromatin Structures of Target LncRNAs in Breast Cancer

**DOI:** 10.3390/ijms252011048

**Published:** 2024-10-15

**Authors:** Zhiyu Fan, Yingli Chen, Dongsheng Yan, Qianzhong Li

**Affiliations:** 1 School of Physical Science and Technology, Inner Mongolia University, Hohhot 010021, China; 22146006@mail.imu.edu.cn (Z.F.); yds015017@163.com (D.Y.); qzli@imu.edu.cn (Q.L.); 2 The State Key Laboratory of Reproductive Regulation and Breeding of Grassland Livestock, Inner Mongolia University, Hohhot 010021, China

**Keywords:** DNA methylation, lncRNA, enhancer, promoter, Hi-C, breast cancer

## Abstract

Aberrant DNA methylation plays a crucial role in breast cancer progression by regulating gene expression. However, the regulatory pattern of DNA methylation in long noncoding RNAs (lncRNAs) for breast cancer remains unclear. In this study, we integrated gene expression, DNA methylation, and clinical data from breast cancer patients included in The Cancer Genome Atlas (TCGA) database. We examined DNA methylation distribution across various lncRNA categories, revealing distinct methylation characteristics. Through genome-wide correlation analysis, we identified the CpG sites located in lncRNAs and the distally associated CpG sites of lncRNAs. Functional genome enrichment analysis, conducted through the integration of ENCODE ChIP-seq data, revealed that differentially methylated CpG sites (DMCs) in lncRNAs were mostly located in promoter regions, while distally associated DMCs primarily acted on enhancer regions. By integrating Hi-C data, we found that DMCs in enhancer and promoter regions were closely associated with the changes in three-dimensional chromatin structures by affecting the formation of enhancer–promoter loops. Furthermore, through Cox regression analysis and three machine learning models, we identified 11 key methylation-driven lncRNAs (DIO3OS, ELOVL2-AS1, MIAT, LINC00536, C9orf163, AC105398.1, LINC02178, MILIP, HID1-AS1, KCNH1-IT1, and TMEM220-AS1) that were associated with the survival of breast cancer patients and constructed a prognostic risk scoring model, which demonstrated strong prognostic performance. These findings enhance our understanding of DNA methylation’s role in lncRNA regulation in breast cancer and provide potential biomarkers for diagnosis.

## 1. Introduction

Breast cancer is among the most prevalent cancers worldwide, and it is a leading cause of cancer-related mortality in women [1]. Therefore, it is crucial to obtain a deeper understanding of the molecular regulatory mechanisms underlying breast cancer to improve its diagnosis, treatment, and prognosis. Long noncoding RNAs (lncRNAs) are a class of transcripts longer than 200 nucleotides with no or limited protein-coding ability [2]. Accumulating evidence has shown that lncRNAs are involved in various biological processes, including cell proliferation, apoptosis, differentiation, tumorigenesis, and metastasis [3,4]. In breast cancer, the expression of certain lncRNAs is significantly altered, influencing tumorigenesis, cell proliferation, and differentiation by regulating gene expression [5]. For instance, the overexpression of kinase-activated long intergenic noncoding RNA (LINC01139) can induce metastatic breast tumors by reducing immune surveillance through the downregulation of antigen presentation by cancer cells [6]. The lncRNA MAGI2-AS3 inhibits breast cancer progression by positively regulating the Fas/FasL signaling pathway [7]. Additionally, lncRNA HOTAIR has been shown to drive cancer metastasis by reprogramming chromatin states [8].

DNA methylation is a key epigenetic modification regulating gene expression and the chromatin structure [9,10,11]. Emerging evidence indicates that epigenetic changes in DNA methylation can affect lncRNA expression patterns and contribute to carcinogenesis. In addition to directly regulating the expression of lncRNAs through their interaction with DNA methylation, recent studies have also demonstrated a more complex regulatory relationship between DNA methylation and lncRNAs [12,13]. For example, hypomethylation of the promoter region of lncRNA SNHG12 leads to its upregulation in gliomas, which in turn promotes the malignant progression of these tumors [14]. The lncRNA EPIST was identified as a tumor suppressor gene in gastric cancer with aberrant hypermethylation near its transcription start site (TSS) linked to its expression and specifically associated with cancer [15]. The lncRNA ESRP2 and ESRP2-as are coordinately expressed from a bidirectional promoter and regulated by the methylation of a proximal enhancer [16].

There is abundant evidence that changes in the three-dimensional chromatin structure not only influence gene expression regulation but also play a significant role in cancer occurrence and progression [17,18]. Consequently, it is important to study three-dimensional chromatin structural changes in cancer and the relationship with gene dysfunction and pathogenesis. For instance, studies demonstrate that in the MCF-7 cell lines with long-term estrogen deprivation, the lncRNA in the active region delimits the topological association domain (TAD) boundary of the ESR1 locus, regulating the apoptosis of breast cancer cells [19]. Additionally, alterations in the three-dimensional genome can disrupt gene expression and lead to disease by remodeling enhancer–promoter interactions. Yang et al. discovered that neo-loops formed through enhancer hijacking regulate the expression of oncogenic transcription factors (including TLX3, TAL2, and HOXA) in T-lineage acute lymphoblastic leukemia patients [20].

In this study, we performed an integrative analysis of DNA methylation data and transcriptome data to explore the relationships between DNA methylation level and lncRNA expression as well as their prognostic value in breast cancer. A comparison with the methylation distributions in protein-coding genes, miRNAs, and lncRNAs revealed several differential methylation signatures. We also analyzed and compared the differences in the methylation distributions of different lncRNA categories. To investigate the main regulatory effects of DNA methylation on lncRNAs, we performed a comprehensive genome-wide correlation analysis combined with ChromHMM segmentation [21]. This allowed us to identify differentially methylated CpG sites (DMCs) that regulate the expression of lncRNAs through promoter and enhancer regions, respectively. In addition, we used Hi-C data to verify the impact of aberrant DNA methylation on the chromatin structure of lncRNAs.

## 2. Results

### 2.1. DNA Methylation Distribution Patterns in lncRNAs

**Figure 1:**
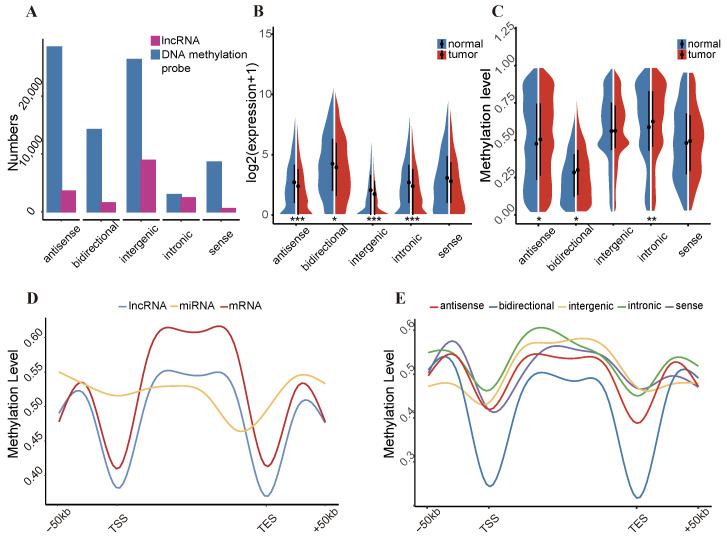
Probe annotation and methylation features of lncRNAs. (**A**) Number of DNA methylation probes and lncRNAs for five lncRNA categories. (**B**) Expression levels of five lncRNA categories across tumor and normal samples. (**C**) Global methylation value distribution of five lncRNA categories across tumor and normal samples. (**D**) DNA methylation patterns of protein-coding genes, lncRNAs, and miRNAs across the gene body and ±5 kb flanking regions of the gene body. (**E**) DNA methylation patterns of five different categories of lncRNAs across the gene body and ±5 kb flanking the gene body. Analyses utilized TCGA-BRCA DNA methylation and gene expression data. Statistical significance was assessed using the *t*-test (* *p* < 0.05, ** *p* < 0.01, *** *p* < 0.001).

This study classified lncRNAs into five categories on the basis of their positions relative to protein-coding genes on the reference genome. Intergenic lncRNAs constitute the largest proportion, accounting for approximately 53% of the total, with 8440 identified instances. Antisense lncRNAs account for 21%, intronic lncRNAs account for approximately 14%, bidirectional lncRNAs account for 9%, and sense lncRNAs account for only 3% (Supplementary Table S1). To characterize the distribution pattern of DNA methylation in lncRNAs, DNA methylation probes were mapped to these lncRNAs. In total, 107,979 methylation probes were mapped to 9773 lncRNA regions. Among these, 35,044 were found in antisense lncRNA regions and 33,628 were found in bidirectional lncRNA regions. Notably, 429 (83.9%) of the 511 sense lncRNA regions were mapped to 8432 methylation probes (Figure 1A). Further analysis revealed significant differences in the expression and methylation levels of these five lncRNAs categories between tumor and normal samples. Specifically, the expression levels of all five lncRNA categories were generally lower in tumor samples. Except for intergenic and sense lncRNAs, tumor samples generally exhibited higher methylation levels, which was consistent with the negative correlation between lncRNA expression and DNA methylation levels in promoter regions (Figure 1B,C). To study the regulatory relationship between lncRNAs and DNA methylation, we first evaluated the DNA methylation distribution patterns flanking the transcription start site (TSS) and transcription termination site (TES) of protein-coding genes, lncRNAs, and microRNAs (miRNAs). The results revealed that protein-coding genes and lncRNAs exhibit similar DNA methylation distribution patterns. In contrast, the DNA methylation levels of miRNAs were relatively stable (Figure 1D). The results revealed disparities in the differential DNA methylation distribution patterns across the five categories of lncRNAs. Compared with other types of lncRNAs, intronic lncRNAs showed a greater enrichment of DNA methylation within the transcribed regions. These findings suggest that different categories of lncRNAs possess unique methylation distribution patterns, which may impact their specific regulatory functions (Figure 1E).

### 2.2. Identification of Key Differential DNA Methylation Regulatory Regions

**Figure 2:**
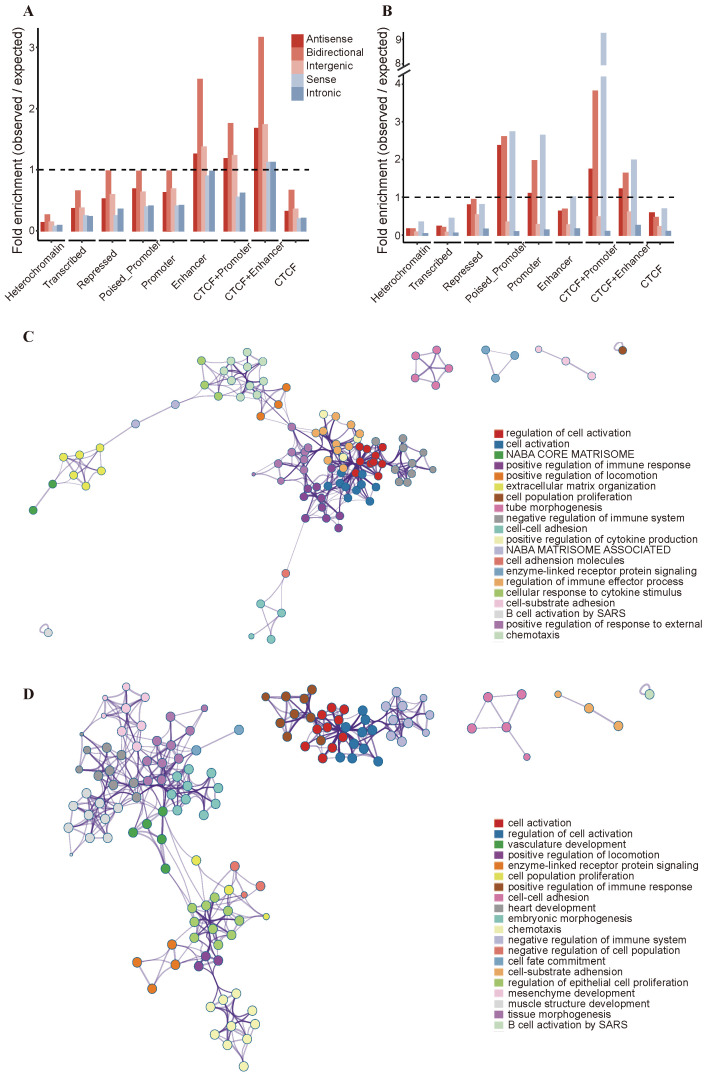
Genomic location of proximal and distally associated CpGs of lncRNAs according to ChromHMM and TF binding regions (**A**) Bar plot showing the enrichment of distally associated CpGs of lncRNAs across functional regulatory regions based on MCF-7 ChromHMM annotation. The bars represent the ratio of observed to expected frequencies for distal CpGs. (**B**) Enrichment of the proximal CpGs of lncRNAs in functional regulatory regions. (**C**) Functional annotation of the lncRNAs with DMCs in the relevant enhancer regions. Top 20 clusters with their representative enriched terms (colored by cluster ID), where nodes that share the same cluster ID are typically close to each other. (**D**) Network plot of the lncRNAs with DMCs in promoter regions. Data obtained from MCF-7 ChromHMM annotation and Metascape analysis.

Current research indicates that DNA methylation can regulate lncRNA expression through acting on lncRNA regions or long-range interactions [22]. To identify long-range interactions between lncRNAs and CpG sites, we correlated DNA methylation levels at non-overlapping CpG sites with the expression levels of lncRNAs on the same chromosome in the TCGA-BRCA cohorts [23]. We identified 37,815 CpG sites that were significantly negatively correlated with 2344 lncRNAs (Bonferroni corrected Spearman correlation *p*-value < 0.05), and these CpG sites were called distally associated CpG sites. To comprehensively explore the functional genomic localization of the distally associated DMCs, the ChromHMM segmentation method was used in the MCF-7 cell lines. We performed enrichemnt analysis in the functional region by the ratio of the frequency of distally associated DMCs in specific segment type to the expected frequency of DMCs from the Illumina HumanMethylation450 array. The results shown that distally associated DMCs are highly enriched in enhancer, CTCF+enhancer and CTCF+promoter regions. The five categories of lncRNAs were more enriched in enhancer, CTCF+enhancer and CTCF+promoter regions compared to other functional regions (Figure 2A). In addition, we investigated the locations of DMCs acting on lncRNA regions and observed that these sites were mainly enriched in poised promoter, promoter, CTCF+promoter, and CTCF+enhancer regions (Figure 2B). These results show that the DMCs of promoter regions and distal enhancer regions play a role in regulating the expression of lncRNAs.

To elucidate the biological relevance of lncRNAs regulated by promoters and enhancers, we utilized the Metascape database to examine the enrichment of functional pathways for lncRNAs with DMCs in their enhancer and promoter regions. The co-enriched pathways included cell activation, immune response regulation, cell population proliferation, cell–cell adhesion, signal transduction, and extracellular matrix organization. Compared to the lncRNAs with DMCs in promoter regions, the lncRNAs with DMCs in enhancer regions were primarily enriched in pathways related to mesenchyme development and morphogenesis, signaling pathways, and cell chemotaxis (Figure 2C). Additionally, the lncRNAs with DMCs in promoter regions were primarily enriched in heart development, embryonic morphogenesis, cell fate commitment, negative regulation, and the regulation of epithelial cell proliferation (Figure 2D).

### 2.3. Identification of Key Methylation-Driven lncRNAs in Breast Cancer

On the basis of the previous results, we focused on the differentially expressed lncRNAs that have at least one DMC within their promoter or associated enhancer regions; these lncRNAs were termed breast cancer-related methylation-driven lncRNAs. To identify the relevant methylation-driven lncRNAs that are closely associated with prognosis, we randomly divided a cohort of 1090 breast cancer patients from the TCGA dataset into a training set (*n* = 763) and a testing set (*n* = 327). Using univariate Cox proportional hazards regression analysis, we identified 42 lncRNAs that were significantly correlated with overall patient survival among the 980 methylation-driven lncRNAs in the training set (*p* < 0.05). Moreover, we utilized three distinct machine learning methodologies to screen for prognostic lncRNAs. The Gradient Boosting Machine (GBM) ranks the top 30 lncRNAs according to their importance. The random forest classifier identified 23 lncRNAs based on ten-fold cross-validation, and LASSO regression analysis identified 27 prognosis-associated lncRNAs. The 11 overlapping lncRNAs (DIO3OS, ELOVL2-AS1, MIAT, LINC00536, C9orf163, AC105398.1, LINC02178, MILIP, HID1-AS1, KCNH1-IT1, and TMEM220-AS1) were commonly identified by these three methods (Supplementary Figure S1 and Table S2).

To evaluate the prognostic value of the 11 key methylation-driven lncRNAs in breast cancer patients, we constructed a risk prediction model by integrating the expression levels of these lncRNAs with the corresponding regression coefficients from the multivariate Cox analysis. The resulting risk score (RS) formula is as follows:

$$\begin{array}{cc} RS= & \;-0.393\times el_{DIO3OS}-0.206\times el_{ELOVL2-AS1}-0.434\times el_{MIAT}+0.292\times el_{LINC00536}\\ \; & \;+0.529\times el_{C9orf163}-6.580\times el_{AC105398.1}+1.533\times el_{LINC02178}-0.305\times el_{MILIP}\\ \; & \;+1.835\times el_{HID1-AS1}-0.984\times el_{KCNH1-IT1}+0.742\times el_{TMEM220-AS1} \end{array}$$

**Figure 3:**
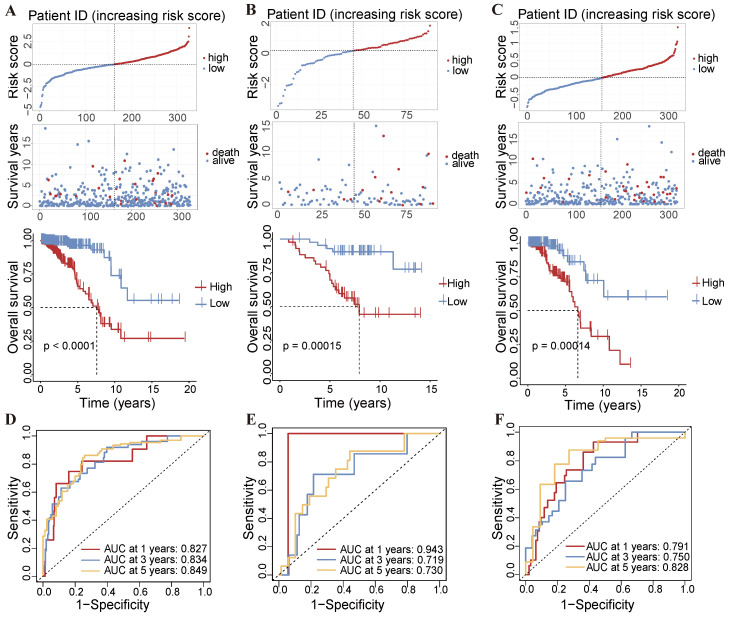
Assessment of prognostic values in the TCGA-BRCA, GSE20711, and GSE20685 datasets. Comparison of the OS status of breast cancer patients with varying risk scores, and K–M curves in (**A**) TCGA-BRCA, (**B**) GSE20711, and (**C**) GSE20685 datasets. The AUC values for the time-dependent ROC curves depict the OS prediction values for the (**D**) TCGA-BRCA, (**E**) GSE20711, and (**F**) GSE20685 datasets. Statistical significance was determined using the log-rank test for K–M curves and the concordance index (C-index) for AUC calculations.

Using this formula, we calculated the risk score for each patient in the TCGA-BRCA dataset. Patients were then stratified into high-risk and low-risk groups based on the median risk score [24]. Our analysis revealed that patients in the high-risk group had significantly shorter overall survival (OS) compared to those in the low-risk group. The Kaplan–Meier survival analysis demonstrated that the high-risk group had a significantly poorer OS (*p* < 0.001) (Figure 3A). Furthermore, the same methodologies were applied to the independent GSE20711 and GSE20685 datasets, and consistent results were obtained (Figure 3B,C). The predictive accuracy of the risk score was evaluated using time-dependent ROC analysis based on the TCGA-BRCA dataset, which revealed areas under the curve (AUCs) of 0.827 for 1 year, 0.834 for 3 years, and 0.849 for 5 years, respectively (Figure 3D). Additionally, in the sets GSE20711 and GSE20685, the AUC values for 1, 2, and 3 years were 0.943, 0.719, and 0.730, and 0.791, 0.750, and 0.828, respectively (Figure 3E,F). These findings indicate the robust prognostic capability of the risk model for breast cancer patients.

**Figure 4:**
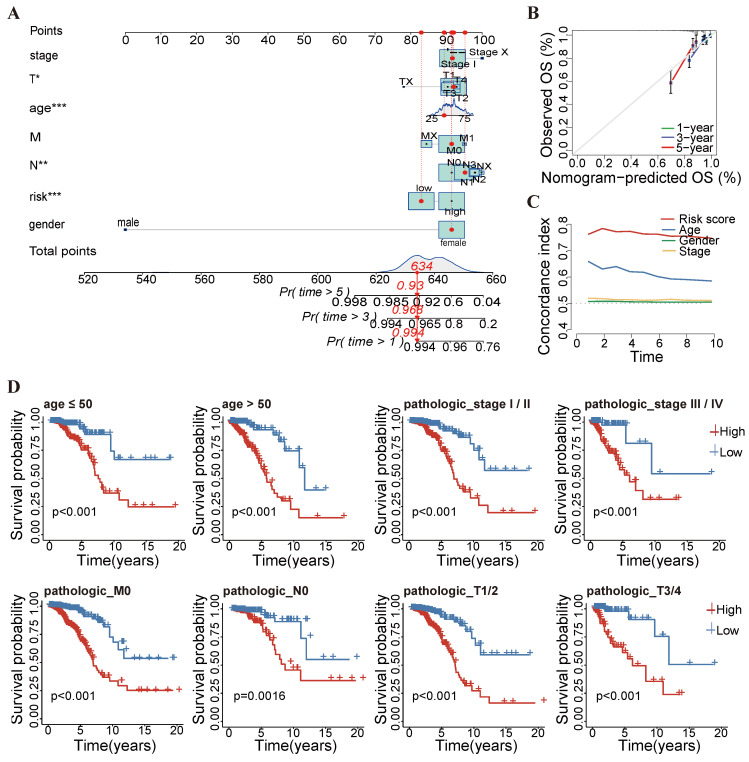
Determination of the independent prognostic value of risk model (**A**) A signature-based nomogram was applied to estimate 1-, 3-, and 5-year overall survival probabilities.(* *p* < 0.05, ** *p* < 0.01, *** *p* < 0.001). (**B**) Calibration plots of the nomogram for predicting the 1-, 3-, and 5-year OS. (**C**) The C-index was utilized to assess and compare the prognostic accuracy between clinical factors and the risk score. (**D**) Subgroup analysis of the Kaplan–Meier (K–M) survival curves was conducted using the log-rank test based on factors such as age, histopathological grade, and clinical stage. All results were based on the TCGA-BRCA dataset.

We developed a nomogram integrating the risk score with clinicopathological characteristics including age, sex, and stage, enabling the prediction of 1-, 3-, and 5-year survival probabilities for breast cancer patients (Figure 4A). Calibration curves demonstrated the reliability of the nomogram in predicting 1-, 3-, and 5-year OS in breast cancer patients (Figure 4B). Furthermore, we observed that the C-index value of the risk score was greater than that of other clinical characteristics, such as age, gender, and stage (Figure 4C). Subsequently, the stratified survival analysis was performed to validate the prognostic performance of the risk score based on clinical characteristics such as age, tumor stage, pathological grade, T category, N category, and M category. Kaplan–Meier curves revealed significant differences in OS between patients in the high-risk and low-risk groups across various clinical subgroups (*p* < 0.05), confirming the consistent predictive performance of the risk score model across subgroups (Figure 4D). Additionally, we performed principal component analysis (PCA) to visualize the distribution of breast cancer patients, and the results revealed that the key methylation-driven lncRNA could help to differentiate the breast cancer patients according to their risk score values (Supplementary Figure S2).

### 2.4. The Effect of DNA Methylation on Three-Dimensional Spatial Structures of LncRNAs

To investigate the regulatory effect of DNA methylation in enhancer and promoter regions on lncRNAs, we utilized whole-genome chromatin conformation capture (Hi-C) data to elucidate the three-dimensional spatial structures of lncRNAs [25,26]. For the methylation-driven lncRNAs in breast cancer, we categorized them into two groups based on the specific positions of DMCs: DP-lncRNAs (DMCs located in the promoter regions of lncRNAs) and DE-lncRNAs (DMCs located in the enhancer regions associated with lncRNAs). For the DP-lncRNAs, we selected 500 kb upstream and 500 kb downstream of the TSS as regulatory regions and calculated the interactions within these regions at 10 kb resolution. We observed specific interactions between these DP-lncRNAs and their differential methylation sites in their respective promoter regions, which displayed pronounced tumor-specific patterns (Supplementary Figure S3). Specifically, our study identified the DP-lncRNA TMEM220-AS1 as a crucial regulator, exhibiting a significantly high expression in the MCF-7 cell lines.

**Figure 5:**
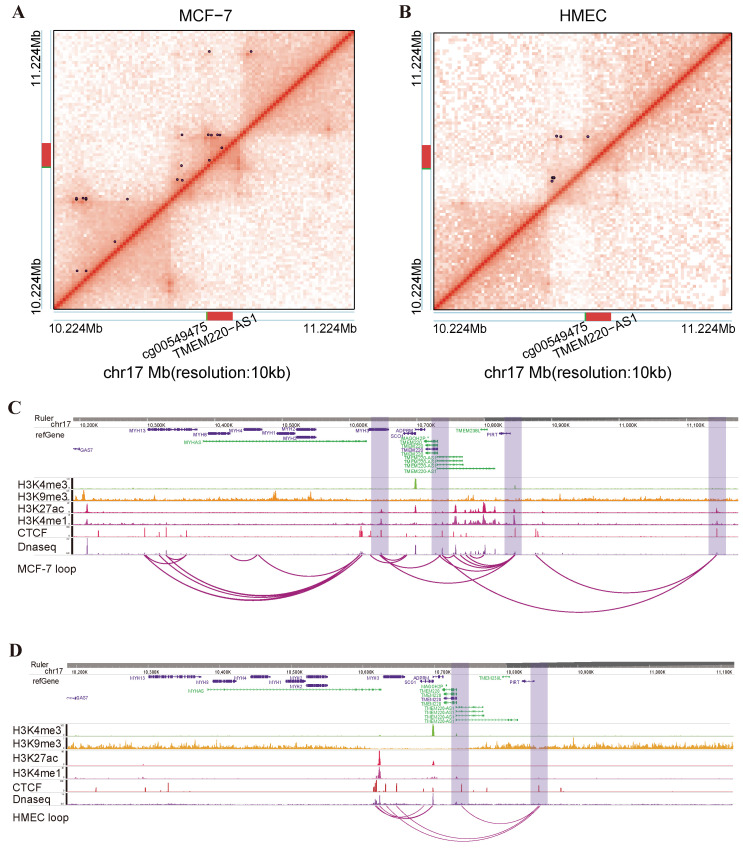
Effect of DMCs on enhancer–promoter looping and DP-lncRNA TMEM220-AS1 expression. Hi-C contact maps for the regions of chromosome 17 (10.224 Mb–11.224 Mb) at 10 kb resolution in the (**A**) MCF-7 cell lines and (**B**) HMEC cell lines; purple points indicate chromatin loops in the HMEC and MCF-7 cell lines, respectively; red rectangles represent TMTM22–AS1; green rectangles indicate significant CpG sites. (**C**) Genome browser snapshots of TMEM220-AS1 in the MCF-7 cell lines, the purple vertical bars highlight the important loop anchor regions which are associated with DP-lncRNAs. The promoter of TMEM220-AS1 co-localize with the anchor of important chromatin loop, DNase-seq peaks, and histone (H3K27ac, H3K4me1) ChIP-seq peaks, and (**D**) genome browser snapshots of TMEM220-AS1 in the HMEC cell lines. Hi-C data were obtained from the ENCODE database, and snapshots were generated using the Washu Epigenome Browser.

A significantly hypomethylated CpG site, cg00549475, is located in the promoter region of TMEM220-AS1 and is involved in three chromatin loops. In HMEC, the expression of TMEM220-AS1 is low, and only one chromatin loop is involved in its promoter region (Figure 5A,B). Typically, the low methylation level of the cg00549475 site could amplify promoter activity, enhancing TMEM220-AS1 expression in the MCF-7 cell lines. Furthermore, in the MCF-7 cell lines, one of the two chromatin loop anchors overlaps with the promoter region of TMEM220-AS1, and the distal regions mapped by the other loop anchor are enriched in H3K27ac and H3K4me1 peaks (Figure 5C,D).

**Figure 6:**
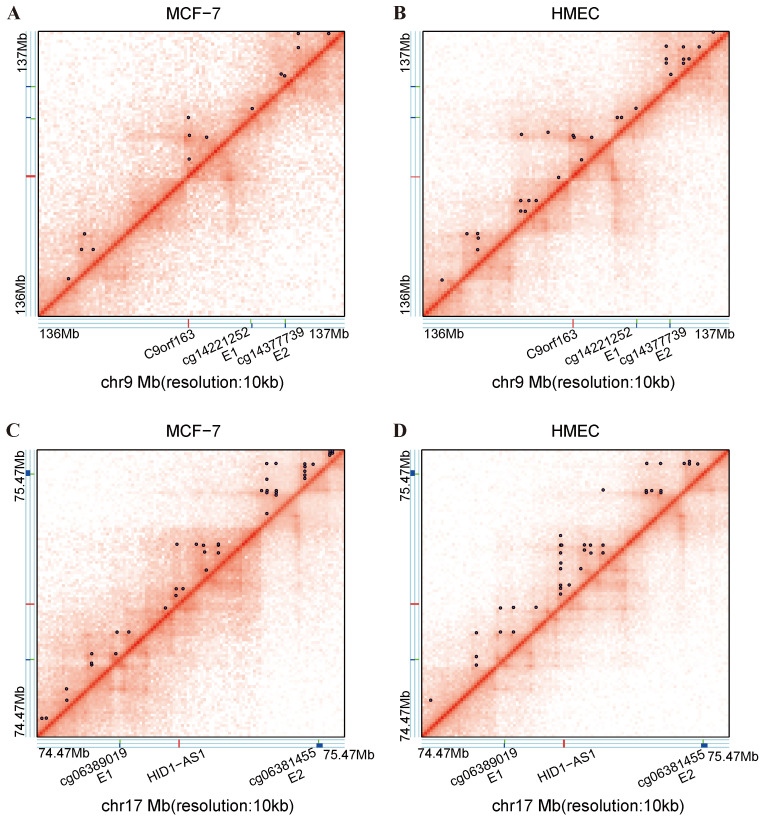
DNA methylation affects 3D genome architecture through distal enhancers. (**A**) Hi-C contact maps of C9orf163 in the MCF-7 cell lines and (**B**) HMEC cell lines. (**C**) Hi-C contact maps of HID1-AS1 in the MCF-7 cell lines and (**D**) HMEC cell lines. Hi-C data were obtained from the ENCODE database. (Purple point: chromatin loops; Red: lncRNAs; Green: significantly different CpG sites, Blue: enhancer regions.).

For the DE-lncRNAs, we focused on exploring how DMCs on enhancers regulate the expression of target lncRNAs through long-range interactions. We identified enhancer elements with DMCs as potential enhancer regulatory regions and analyzed chromatin interactions within this region at 10 kb resolution (Supplementary Figure S4). In the MCF-7 cell lines, we identified a cell-type specific chromatin loop: one anchor was located in the promoter region of C9orf163, and another anchor was overlapping with the enhancer region of C9orf223 that was identified by the distal regulatory DMC cg14221252. However, such chromatin loops were not observed in the HMEC cell lines (Figure 6A,B). This suggests that the spatial interaction between the DE-lncRNAs and the enhancers are regulated by their associated CpG sites. In the HMEC cell lines, the distally associated hypomethylated CpG site cg06389019 was located in the enhancer region of HID1-AS1. We observed that one loop anchor was located near the promoter of HID1-AS1, whereas another loop anchor overlapped with the enhancer acted upon by cg06389019. This spatial interaction between the enhancer and promoter promoted a high expression of HID1-AS1 in the HMEC cell lines. In contrast, in the MCF-7 cell lines, the hypermethylation of cg06389019 may hinder the formation of enhancer–promoter interactions, resulting in lower expression levels of HID1-AS1 (Figure 6C,D).

### 2.5. Correlations between TME, Immune Cell Infiltration Levels and Risk Scores

**Figure 7:**
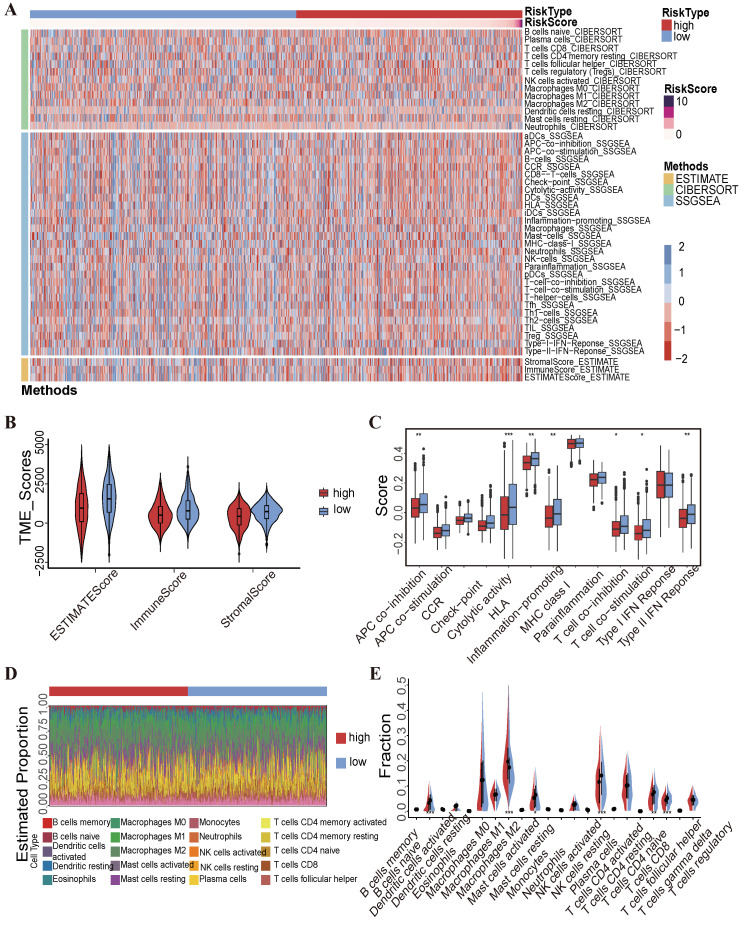
Relationships between immune cell infiltration, the TME, and the key methylation-driven lncRNA signature. (**A**) Heat map of immune responses among the high- and low-risk groups based on the CIBERSORT, ESTIMATE, and ssGSEA algorithms. (**B**) Comparison of TME scores in both risk groups via the ESTIMATE algorithm. (**C**) Box plot comparing 13 immune-linked functions in both risk groups. (**D**) The CIBERSORT algorithm was used to quantify the distribution of 22 tumor-infiltrating immune cells in all HNSCC patients. (**E**) Violin plot showing the fraction of 22 immune cells in both risk groups. Statistical significance was assessed using the *t*-test (* *p* < 0.05, ** *p* < 0.01, and *** *p* < 0.001).

In this study, we used three algorithms to evaluate the association between TME status and risk scores in breast cancer patients. The results revealed that low-risk patients have significantly higher immune scores compared to high-risk counterparts (Figure 7A,B). Utilizing single-sample gene set enrichment analysis, we found that low-risk patients were characterized by the greater enrichment of APC co-inhibition, cytolytic activity, human leukocyte antigen (HLA), inflammation-promoting, T cell co-inhibition, Type II IFN response, and T cell co-stimulation pathways (Figure 7C). These findings suggest that they have an enhanced immune response and potentially better prognosis in low-risk patients. Additionally, the CIBERSORT algorithm was used to quantify the relative proportions of 22 types of tumor-infiltrating immune cells in each patient and correlate these proportions with risk scores (Figure 7D). The low-risk patients presented greater infiltration levels of naive B cells, resting mast cells, plasma cells, CD8+ T cells, and follicular helper T cells compared with high-risk patients (Figure 7E). These findings suggest that low-risk patients have a more active and diverse immune environment, which may contribute to their better prognosis.

## 3. Discussion

In this study, we have discussed the molecular mechanisms between lncRNA and genome-wide DNA methylation found in breast cancer. Compared with protein-coding genes, lncRNAs exhibit overall lower levels of methylation, and their methylation distribution demonstrates a clear type-specificity, indicating a unique role for lncRNAs in the epigenetic regulation of breast cancer. By the comparison of the genome-wide correlation analysis of lncRNA transcriptome and DNA methylation, we found that the DMCs which regulated lncRNA expression are mostly located in the enhancer or promoter regions. DNA methylation is associated with less accessible regions of enhancers, directly prevents the binding of some transcription factors [27,28], decreases enhancer–promoter proximity and reduces gene expression [29]. Kang et al. discovered that the de novo methylation of CpG islands in the promoter regions is an epigenetic mark of gene silencing in cancer; DNA methylation disrupts the proper chromatin loops of the PTGS2 locus by limiting the binding of CTCF/cohesin to the PTGS2 locus, which subsequently reduces the PTGS2 expression [30]. Our study revealed the close correlation between the DMCs in enhancer and promoter regions and the changes in the chromatin structure of target lncRNAs. In the MCF-7 cell lines, the hypomethylated CpG site cg00549475 located in the promoter of TMEM220-AS1 contributed to the formation of enhancer–promoter loops, and the hypomethylated CpG site cg14221252 located in the enhancer of C9orf163 contributed to the formation of enhancer–promoter loops.

This study identified 11 key methylation-driven lncRNAs (DIO3OS, ELOVL2-AS1, MIAT, LINC00536, C9orf163, AC105398.1, LINC02178, MILIP, HID1-AS1, KCNH1-IT1, and TMEM220-AS1) closely associated with breast cancer prognosis through three machine learning methods. Several of these lncRNAs have been found to be related to the pathogenesis and advancement of the disease, as indicated by correlation studies. For instance, the overexpression of ELOVL2-AS1 can inhibit the migration of triple-negative breast cancer cells [31]. MIAT is overexpressed in breast cancer, and its inhibition triggers senescence and G1 arrest in MCF7 cell lines [32,33]. The silencing of LINC00536 inhibits the proliferation, migration, and invasion of breast cancer cells [34].

Furthermore, we evaluated the predictive ability of the prognostic model constructed from the 11 key methylated lncRNAs. In the TCGA-BRCA dataset, the model achieved AUC values of 0.827 (1 year), 0.834 (3 years), and 0.849 (5 years). We searched for recent studies on lncRNA prognostic signatures based on TCGA-BRCA data. The AUC of the mutator-derived lncRNA prognostic model by Bao et al. was 0.747 [35], while Li et al. reported an AUC of 0.813 for the stemness-related lncRNA prognostic model [36]. Additionally, Wang et al.’s arginine methylation-associated lncRNA model had an AUC of 0.777 [37]. These results demonstrate the superiority of our methylation-driven lncRNA model for long-term prognostic prediction. Although many studies have evaluated different lncRNA combinations as biomarkers for breast cancer prognosis, the uniqueness of our model lies in its integration of epigenetic dimensions. It emphasizes the regulatory role of DNA methylation in the expression of lncRNAs at enhancer and promoter regions, which has significant implications for breast cancer prognosis.

Early studies had shown that high immune cell infiltration is strongly linked to better prognosis in breast cancer patients [38,39,40]. Our results show that low-risk breast cancer patients have significantly higher immune scores and the increased infiltration of immune cells like CD8+ T cells, follicular helper T cells, and plasma cells, which indicated a more active immune response in the tumor microenvironment. The enhanced immune response likely contributes to stronger anti-tumor activity. Key immune pathways for effective anti-tumor immune responses were also enriched, including antigen-presenting cell (APC) co-inhibition, cytolytic activity, and T-cell co-stimulation. Therefore, low-risk patients may have a more favorable immune-mediated tumor microenvironment, leading to better prognoses. These findings emphasize the role of immune response in breast cancer risk assessment and prognosis, offering potential research directions for personalized immunotherapy.

This study elucidates the regulatory mechanism of DNA methylation on lncRNA in breast cancer and identifies key prognostic signatures prediction; however, it has limitations that require further exploration. First, although the model showed robustness in the TCGA and two GEO datasets, more independent external datasets are needed to validate its generalizability. Additionally, this study is primarily based on the MCF-7 cell line, which is the luminal A subtype, and it has not covered all molecular subtypes of breast cancer. Consequently, future research should include more breast cancer subtypes to comprehensively evaluate the role of DNA methylation in regulating lncRNA expression through a three-dimensional chromatin structure in different subtypes and further validate the broad applicability of this mechanism.

## 4. Materials and Methods

### 4.1. Multi-Omics Data Acquisition and Processing

The DNA methylation data (Illumina Infinium HumanMethylation 450K), gene expression data, and clinical data for breast cancer and adjacent normal tissue samples were downloaded from The Cancer Genome Atlas database (TCGA; https://portal.gdc.cancer.gov (accessed on 18 November 2023)) [41]. The gene expression data comprised 1231 samples, with 113 normal and 1118 cancer tissue samples; the DNA methylation data comprised 889 samples, with 135 normal and 754 cancer tissue samples. We processed the DNA methylation data using the R package ChAMP (v2.32.0) [42,43]. Initially, probes with missing values in more than 10% of the samples were removed. Subsequently, cross-reactive probes, probes targeting SNPs, and those located on sex chromosomes were excluded. Finally, 412,481 of the original 485,577 CpG sites were retained for further analysis. Missing values were imputed using the k-nearest neighbors (KNN) algorithm, and beta values were normalized to reduce batch effects and ensure comparability between samples. For the gene expression data, lowly expressed genes were filtered out by excluding those with expression values lower than 1 in more than 80% of the samples, and the data were normalized to FPKM (Fragments Per Kilobase of transcript per Million mapped reads) format. Additionally, the gene expression data and clinical data of the validation cohort GSE20685 (327 samples) [44] and GSE20711 (90 samples) [45] from Gene Expression Omnibus (GEO, https://www.ncbi.nlm.nih.gov/geo/ (accessed on 20 February 2024)) were downloaded to validate the ability of our prognostic model. The lncRNA annotation data were obtained from the GENCODE database (GENCODE; https://www.gencodegenes.org/human/ (accessed on 19 November 2023), GRCh38 version) [46]. The Chromatin Immunoprecipitation sequencing (ChIP-seq) data and Hi-C data for the MCF-7 cell lines and human mammary epithelial cells (HMECs) were downloaded from the ENCODE project (ENCODE; https://www.encodeproject.org/ (accessed on 10 January 2024)) (Supplementary Table S3).

### 4.2. Classification of lncRNAs

**Figure 8:**
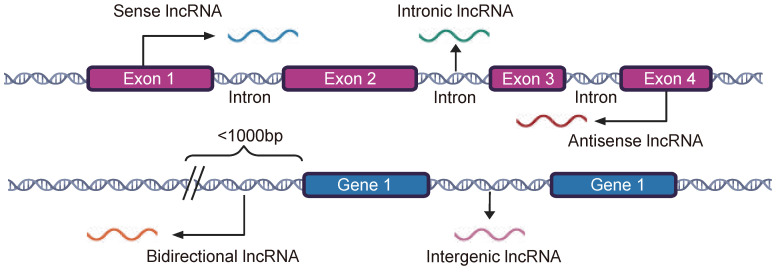
Classification of lncRNAs based on their genomic proximity to neighboring transcripts [50].

The lncRNA annotation files of the human reference genome GRCh38 were downloaded from GENCODE, and 16,901 transcripts annotated as lncRNAs were screened out for analysis. Using BEDtools (v2.29.2) [47], we categorized lncRNAs into five categories based on their genomic positions relative to protein-coding genes [48,49]: (a) sense lncRNAs are defined that overlap with one or more exons of the protein-coding gene on the same strand; (b) antisense lncRNAs are characterized by overlapping exons of one or more protein-coding genes on opposite strands; (c) bidirectional lncRNAs initiate its expression in close genomic proximity within 1000 base pairs away from the neighboring coding transcript on the opposite strand; (d) intronic lncRNAs are defined as those that are located within the introns of protein-coding genes; and (e) intergenic lncRNAs are characterized as an independent unit within the genomic interval between two genes (Figure 8).

### 4.3. Differentially Methylated CpG Sites (DMCs) and Differentially Expressed Genes (DEGs)

To explore the regulatory patterns of DNA methylation in different gene types and different categories of lncRNAs, the gene body regions were divided into 100 bins. Additionally, the 5 kb region upstream of the gene transcription start site and the region downstream of the gene transcription termination site were evenly divided into 50 bins, respectively. The CpG sites were mapped to these 200 bins. We mapped the CpG sites into each bin to calculate the DNA methylation levels. The average levels of methylation for all the samples were calculated using custom R scripts. The average levels of methylation $\beta_{k}$ for the *k*-th bin in different regions were calculated by using Equation (1):

(1) $$\beta_{\omega }=\frac{\sum_{s=1}^{m}\beta_{\omega ,s}}{m}\;\beta_{i}=\frac{\sum_{\omega =1}^{n_{i}}\beta_{\omega }}{n_{i}}\;\beta_{k}=\frac{\sum_{i=1}^{N}\beta_{i}}{N}\;\left(1\leq \omega \leq 412,481,\;1\leq i\leq N\right)$$

where *s* is the *s*-th sample, *m* is the total number of samples, ω is the ω-th CpG sites, and *i* is the *i*-th gene. $\beta_{\omega ,s}$ is the methylation level of the ω-th CpG site in the *s*-th sample. $\beta_{\omega }$ is the average methylation level of the ω-th CpG site in all samples, $n_{i}$ is the number of all CpG sites matching the specific region of the *i* gene, $\beta_{i}$ is the average methylation level in a specific region on gene *i*, and N represents the number of all effective methylated genes with non-zero.

We conducted differential methylation analysis using the ChAMP package (v2.32.0). The threshold for the methylation value of CpG sites was set as $\left|\Delta \beta |=\right|\beta_{\mathrm{Tumour}}-\beta_{\mathrm{Normal}}|>0.2$ and adjusted *p* value <0.01 between the tumor and adjacent normal tissue; Δβ>0.2 was defined as hypermethylated sites, and Δβ<−0.2 was defined as hypomethylated sites.

The DEseq2 package was used to identify differentially expressed genes between tumor tissues and normal tissues with pvalue<0.05 and $|\log_{2}\left(\mathrm{fold}\;\mathrm{change}\right)|>2$ as the threshold values. The fold change (FC) was calculated as Equation (2):

(2) $${\mathrm{FC}}_{i}=\frac{\left(\sum_{j=1}^{n_{T}}x_{ij,T}\right)/n_{T}}{\left(\sum_{j=1}^{n_{N}}x_{ij,N}\right)/n_{N}}$$

where *i* represents the *i*-th gene, *j* represents the *j*-th sample, *T* denotes cancer tissue, and *N* denotes adjacent non-cancerous tissue. $x_{ij,T}$ represents the normalized expression value of the *i*-th gene in the *j*-th cancer (or adjacent non-cancerous) tissue sample. ${\mathrm{FC}}_{i}$ indicates the fold change of the *i*-th gene. $n_{T}$ ($n_{N}$) represents the total number of breast cancer (or adjacent non-cancerous) tissue samples.

### 4.4. Identifying Distally Associated CpG Sites for lncRNAs

We selected CpG sites with an interquartile range >0.1 and analyzed only non-overlapping lncRNAs and CpGs on the same chromosome. The Spearman correlation between the CpG sites and the lncRNAs were calculated using Equation (3) with custom R scripts:

(3) $$P_{uv}=1-\frac{6\sum_{i=1}^{m}\left[{\left(\mathrm{rg}\left(x_{iu}\right)-\mathrm{rg}\left(\beta_{iv}\right)\right)}^{2}\right]}{m(m-1)}$$

where *m* is the sample numbers, $x_{iu}$ denotes the expression levels of the *u*-th lncRNA in the *i*-th sample and $\beta_{iv}$ denotes the DNA methylation levels of the *v*-th CpG loci in the *i*-th sample. $\mathrm{rg}(x_{iu})$ and $\mathrm{rg}(\beta_{iv})$ are the rank numbers of $x_{iu}$ and $\beta_{iv}$, respectively.

### 4.5. Genomic Segmentation and Annotation

To comprehensively characterize the functional genomic location of the CpG sites regulating lncRNAs in breast cancer, we used the ChromHMM (v1.24) segmentation of the MCF-7 genome obtained from Taberlay et al. [51]. Based on ChIP-seq data of key histone modifications (H3K4me2, H3K4me3, H3K9ac, H3K27ac, H3K27me3, H3K4me1, H4K20me1, H3K36me3) and regulatory factors (CTCF, RNA Pol II), we used a multivariate hidden Markov model to annotate the MCF-7 genome into nine distinct chromatin states: heterochromatin, repressed, transcribed, enhancer, enhancer+CTCF, CTCF, promoter+CTCF, promoter, and promoter_poised [52].

### 4.6. Identification of the Key Methylation-Driven lncRNAs through Machine Learning

Patient and normal samples were randomly divided into training and testing sets using the createDataPartition function from the caret package (v6.0.94) [53]. A 70% proportion of the dataset was allocated to the training set, and the remaining 30% was used as the testing set. The partitioning was stratified to ensure a balanced distribution of survival status between the two sets. The Gradient Boosting Machine (GBM) was implemented through the gbm (v2.1.9) package in R [54]. All prognostic-related methylation-driven lncRNAs were then verified by random forest in 10-fold cross-validation using the caret package (v6.0.94). LASSO analysis was carried out by using the glmnet function of the R package glmnet (v4.1-8) to determine the optimal value of the penalty coefficient lambda, and 10-fold cross-validation was performed by using the cv.glmnet function [55].

### 4.7. Survival Analysis

Multivariate Cox proportional hazard regressions were constructed to assess the relationships between the expression levels of the key methylation-driven lncRNAs and survival rates. The hazard ratio (HR) and 95% confidence interval (CI) were calculated. Based on the coefficients of multivariate Cox analysis (calculated using the survival package in R, v3.5.7) and the expression levels of the key methylation-driven lncRNAs, a prognostic risk scoring (RS) model was constructed, and the formula is as follows:

(4) $$\mathrm{RS}=\sum_{l=1}^{M}\left({\mathrm{coef}}_{l}\times {\mathrm{el}}_{l}\right)$$

where *l* denotes the *l*-th lncRNA, *M* denotes the total number of lncRNAs, ${\mathrm{coef}}_{l}$ denotes the regression coefficient and represents the contribution of the *l*-th lncRNA to the prognostic risk score in the multivariate Cox analysis, and ${\mathrm{el}}_{l}$ denotes the expression level of the *l*-th lncRNA.

### 4.8. Comprehensive Assessment of the Prognostic Value of the Risk Model

Scatter plots and risk curves were used to describe the distribution and survival status of the risk scores for all breast cancer patients. The difference in the overall survival (OS) rate between the two risk groups is presented via Kaplan–Meier (K–M) curves. We generated time-dependent receiver operating characteristic (ROC) curves with the risk model to determine the 1-, 3-, and 5-year OS rates using the timeROC (v0.4) and survivalROC (v1.0.3.1) R packages with data from the training, validation, entire cohorts, and external validation sets, respectively [56]. To comprehensively ascertain the efficacy of the risk model, the entire cohort was employed for subsequent evaluation. The Rtsne (v0.17) packages were used to carry out principal component analysis (PCA) and assess whether the whole genome, methylation regulation-related genes, methylation-driving lncRNAs, and risk lncRNAs could categorize the breast cancer patients into high- and low-risk groups. The prognostic efficacy of age, grade, stage, and risk model were compared by using multivariate ROC curves, C-index analysis, and decision curves, which were implemented with the pROC (v1.18.5) [57] and survcomp (v1.52.0) R packages [58]. R packages rms (v6.8.0) were used to create calibration plots and evaluate the predictive performance of the proposed nomogram for patient survival.

### 4.9. Distinct 3D Genome Structure of the Methylation-Driven lncRNAs between the HMEC and MCF-7 Cells

We downloaded Hi-C data .hic files of the HMEC and MCF-7 cell lines from the ENCODE database, which included raw and Vanilla-Coverage normalized (VC) counts, as well as normalization vectors of contact frequency matrices. This method utilized raw and Vanilla-Coverage normalized counts, as well as normalization vectors of contact frequency matrices via the python package HicExplorer (v3.0) at 10 kb resolution. To view chromatin interactions surrounding regulatory regions, we extracted chromatin interaction counts from the VC normalized Hi-C contact matrices [59]. Loops were called at resolutions of 1 kb, 2 kb, and 5 kb with HiCCUPS using default parameters and scaled to 5 kb resolution [60]. To identify significant chromatin interactions and long-range chromatin loops, we filtered the chromatin loops at FDR < 0.05 and the distances between loop anchors greater than 20 kb. To investigate the relationships between the 3D genome and epigenome, we downloaded the histone modification and DNase-seq bigwig files and visualized them in the WashU Epigenome Browser (https://epigenomegateway.wustl.edu/browser/ (accessed on 12 February 2024)) [61].

### 4.10. Tumor Microenvironment (TME) and the Infiltration Levels of the Immune Cells

ESTIMATE, CIBERSORT, and single-sample gene set enrichment analysis (ssGSEA) algorithms were applied using R packages estimate (v1.0.13), CIBERSORT (v0.1.0) [62], and GSVA (v1.50.1) [63] to calculate the abundance of infiltrating immune cells between the high- and low-risk groups [64]. Furthermore, the enrichment scores of 13 immune-related pathways for each breast cancer sample were compared by using the ssGSEA process. CIBERSORT analysis was used to calculate the relative proportions of 22 tumor-infiltrating immune cells in the two risk groups.

### 4.11. Statistical Analysis

Statistical analyses were performed using R software (version 4.3.2). Continuous or categorical variables were compared between the two groups by the *t*-test (normal distribution), respectively. *p* < 0.05 was considered statistically significant. To control for false discovery rate due to multiple comparisons, the Benjamini–Hochberg procedure was applied, and adjusted *p*-values were reported.

## 5. Conclusions

In summary, by integrating multi-omics data, we revealed the association between abnormal DNA methylation, lncRNA expression, and chromatin structures. Additionally, we screened out key methylation-driven lncRNAs that impact breast cancer prognosis. Our research provides new insights into the regulation of DNA methylation in lncRNAs, which will significantly contribute to the understanding of breast cancer mechanisms in the future.

## Data Availability

All the data for this study were obtained from publicly available databases such as TCGA (https://portal.gdc.cancer.gov/, accessed on 9 August 2023) and GEO (https://www.ncbi.nlm.nih.gov/geoprofiles/, accessed on 9 August 2023).

## Supplementary Materials

The following supporting information can be downloaded at: https://www.mdpi.com/article/10.3390/ijms252011048/s1.

## Abbreviations

The following abbreviations are used in this manuscript:

lncRNA	long noncoding RNA
DMC	differentially methylated CpG site
APC	antigen-presenting cell
KNN	k-nearest neighbors
DEG	differentially expressed gene
TSS	transcription start site
TES	transcription termination site
3D	three-dimensional
VC	Vanilla-Coverage normalized
RNA-seq	RNA sequencing
HMEC	human mammary epithelial cell
ChIP-seq	Chromatin Immunoprecipitation sequencing
BH	Benjamini-Hochberg
GBM	gradient boosting machine
OS:	overall survival
K–M	Kaplan–Meier
ROC	receiver operating characteristic
ssGSEA	single-sample gene set enrichment analysis
AUC	area under the curve
PCA	principal component analysis
DP-lncRNAs	DMCs are located in the promoter regions of lncRNAs
DE-lncRNAs	DMCs are located in the enhancer regions associated with lncRNAs
