# Supplementary material for: Effects of Differentially Methylated CpG Sites in Enhancer and Promoter Regions on the Chromatin Structures of Target LncRNAs in Breast Cancer
Source: Int J Mol Sci. 2024 Oct 15;25(20):11048. doi: 10.3390/ijms252011048 (PMC11507307; doi:10.3390/ijms252011048)
Supplement: Supplementary file 1 [file ijms-25-11048-s001.zip › Supplementary Figure.pdf]

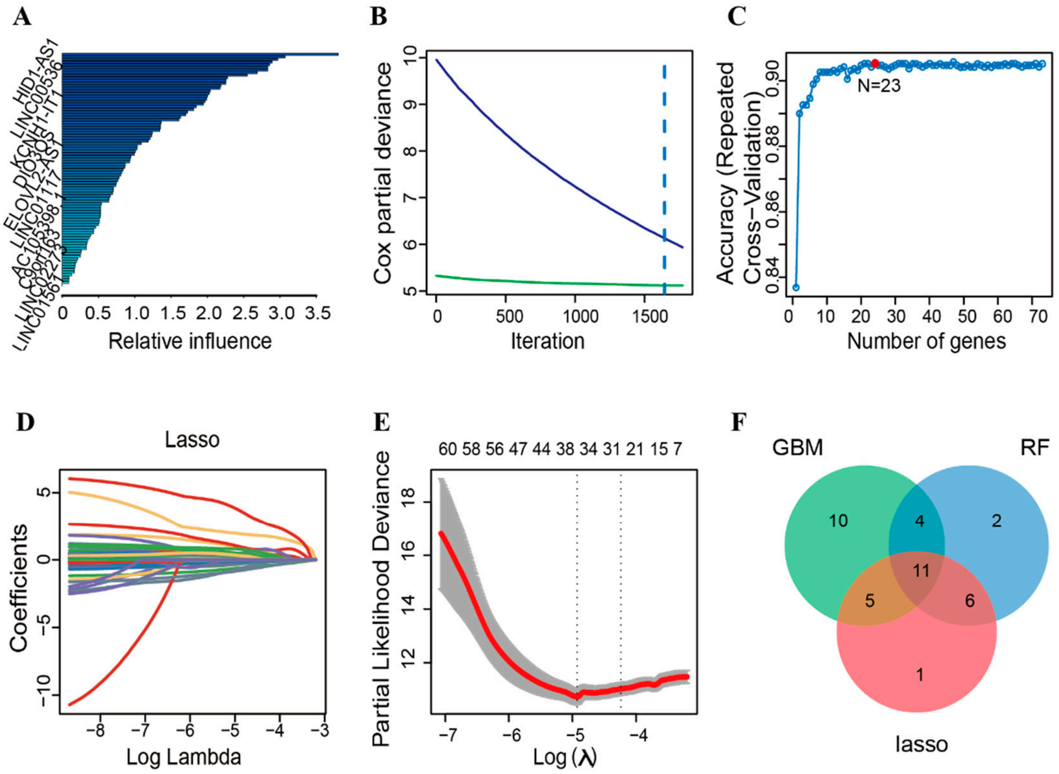

Figure S1. Detection of key methylation-driven lncRNAs using a thorough method. A, B. based on GBM to screen key methylation-driven lncRNAs. C. based on RF algorithm to screen key methylation-driven lncRNAs. D. Different colors represent different lncRNAs; E. LASSO logistic regression algorithm to screen diagnostic markers. F. Venn diagram of key methylation-driven lncRNAs obtained by three machine learning models. GBM: gradient boosting machine, RF: random forest, LASSO: least absolute shrinkage and selection operator.

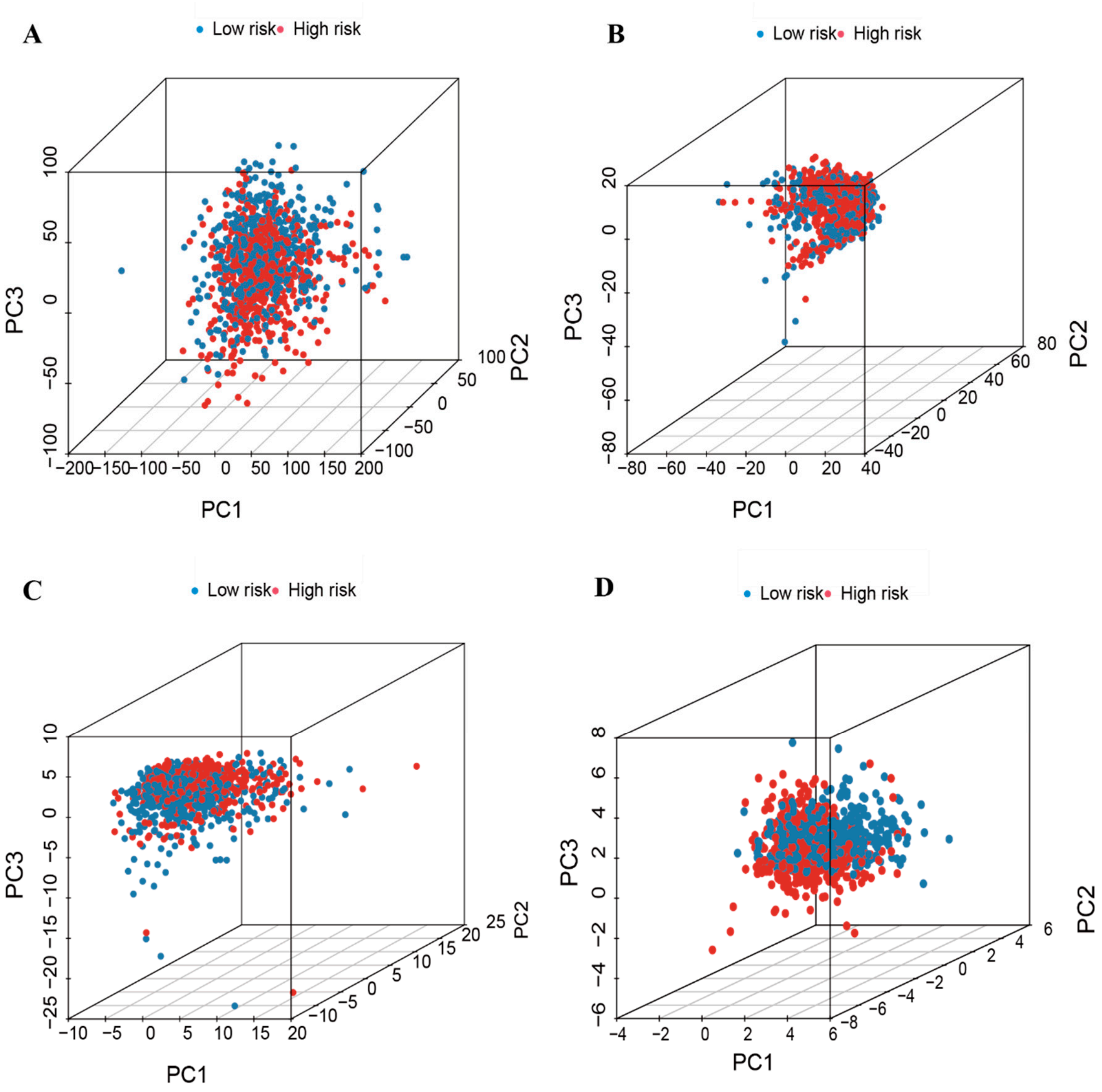

Figure S2. PCA analysis of the data derived from the entire cohort. The results of the PCA analyses indicated that D. the key methylation-driven lncRNAs signature could be differentiated based on the risk status of BC patients compared to the A. whole genome, B. methylation-driven genes and C. methylation-driven lncRNAs.

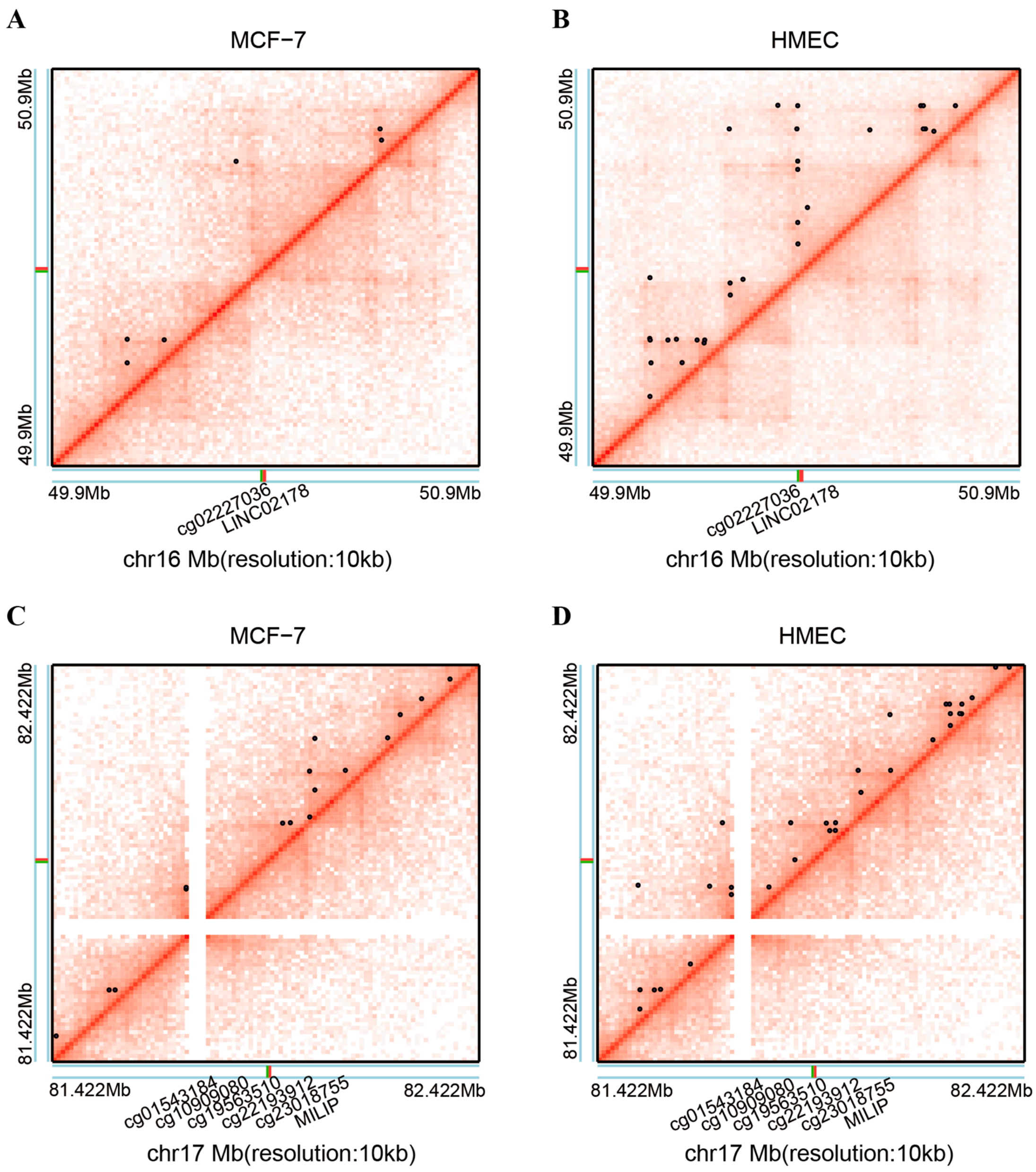

Figure S3. The three-dimensional genome information of the key methylation-driven lncRNAs which occur differential methylation in the promoter regions. Hi-C contact maps of LINC02178 in A. MCF-7 cell line and B. HMEC cell line. Hi-C contact maps of MILIP in C. MCF-7 cell line and D. HMEC cell line. purple point indicates chromatin loops in MCF-7 and HMEC, respectively; red rectangle represents lncRNAs; green rectangle indicates significant CpG sites. cg02227036, cg01543184, cg10909080, cg19563510, cg22193912 and cg23018755 are hypermethylated CpG sites.

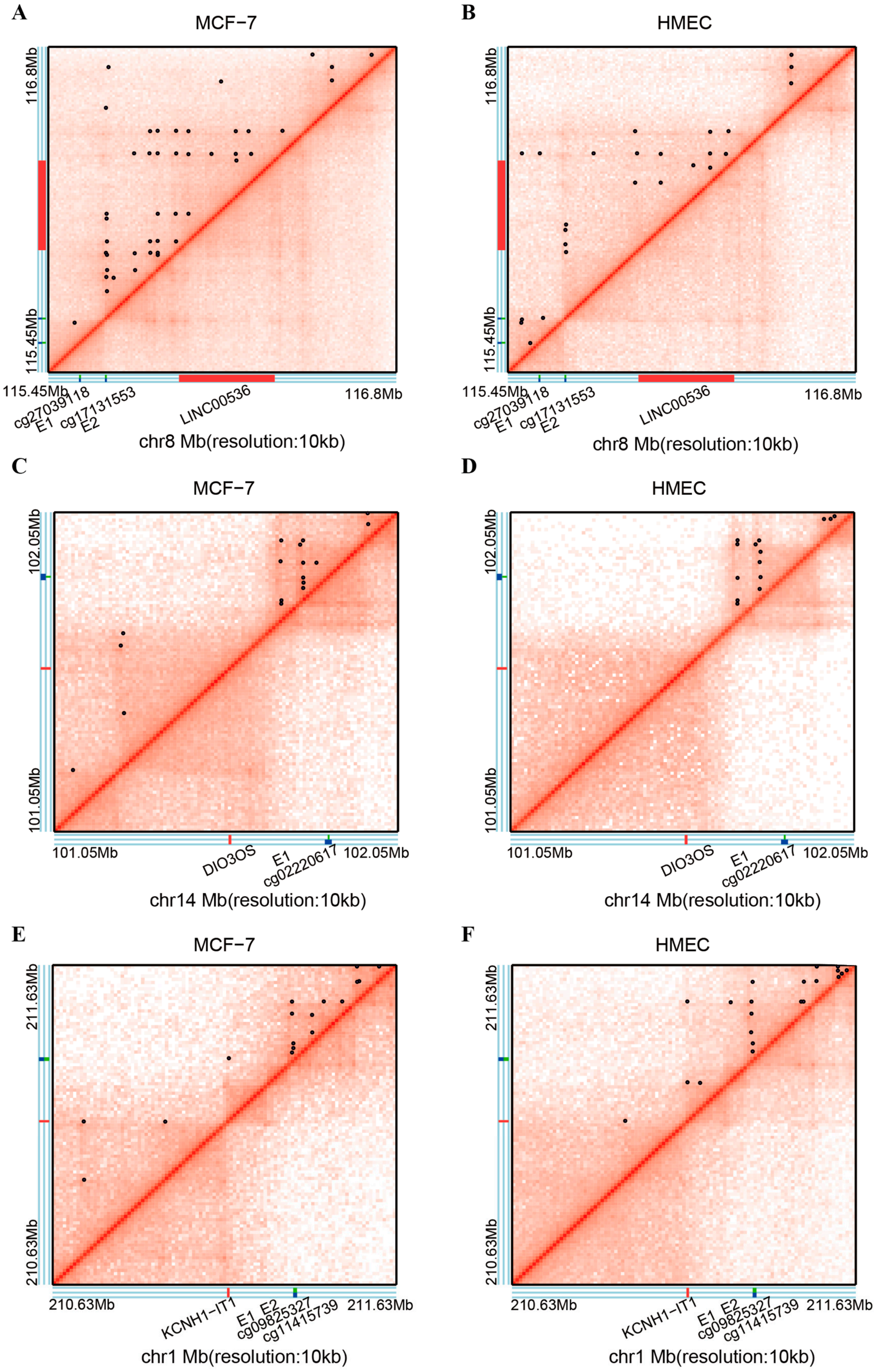

Figure S4. The three-dimensional genome information of the key methylation-driven lncRNAs

which occur differential methylation in the enhancer regions. Hi-C contact maps of LINC00536 in A. MCF-7 cell line and B. HMEC cell line. Hi-C contact maps of DIO3OS in C. MCF-7 cell line and D. HMEC cell line. Hi-C contact maps of KCNH1-IT1 in C. MCF-7 cell line and D. HMEC cell line. purple point indicate chromatin loops in MCF-7 and HMEC, respectively; red rectangle represents lncRNAs; green rectangle indicate significant CpG sites; blue rectangle indicates enhancer regions. cg09825327, cg27039118, cg17131553 and cg11415739 are hypomethylated CpG sites; cg02220617 is hypermethylated CpG site.
