# Supplementary figures and images for: Effects of Differentially Methylated CpG Sites in Enhancer and Promoter Regions on the Chromatin Structures of Target LncRNAs in Breast Cancer
Source: Int J Mol Sci. 2024 Oct 15;25(20):11048. doi: 10.3390/ijms252011048 (PMC11507307; doi:10.3390/ijms252011048)

**A**

● Low risk ● High risk

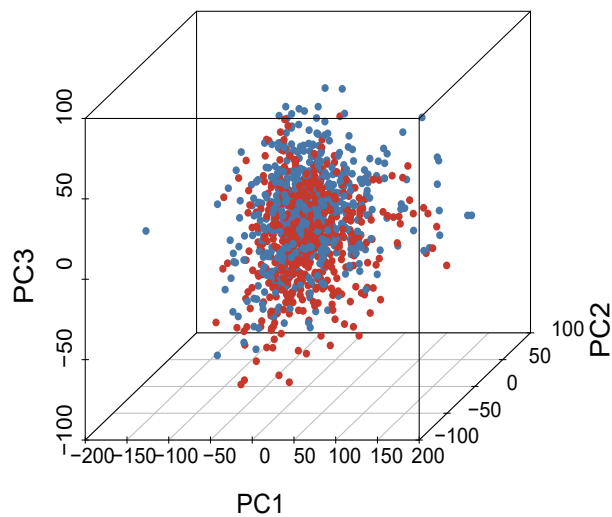**B**

● Low risk ● High risk

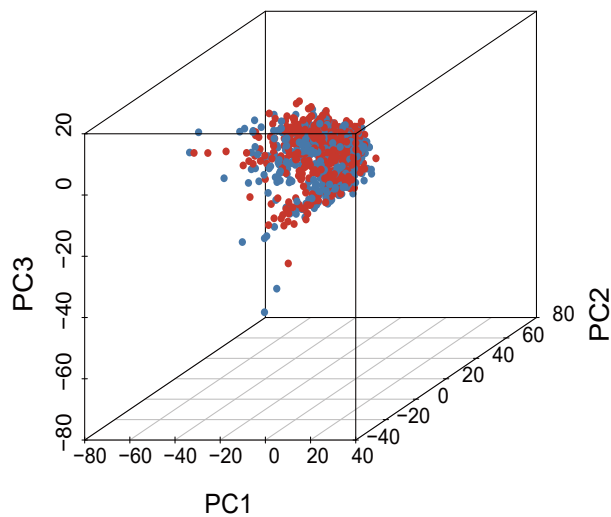**C**

● Low risk ● High risk

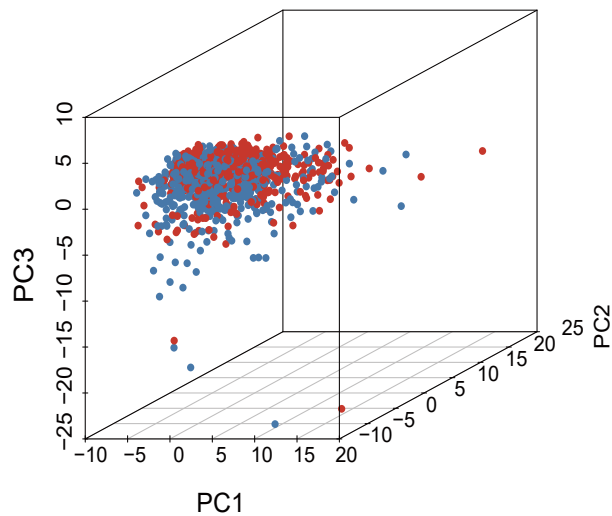**D**

● Low risk ● High risk

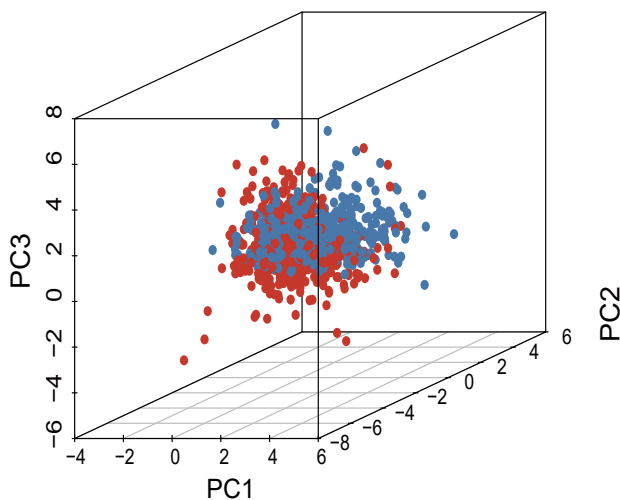

Supplement: Supplementary file 1 [file ijms-25-11048-s001.zip › Figure S2.pdf]

**A****MCF-7**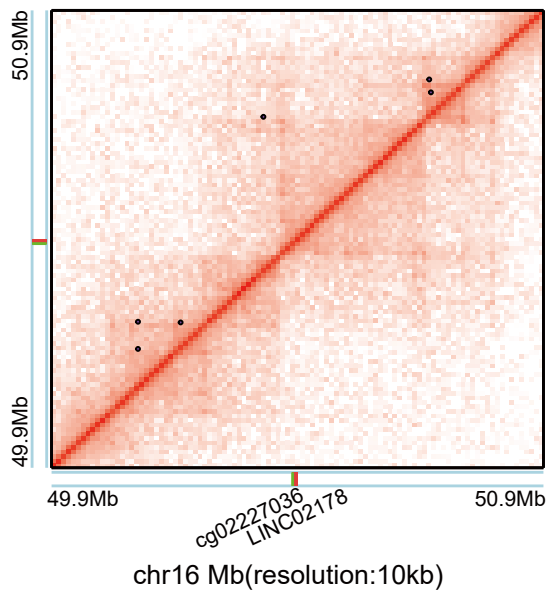**B****HMEC**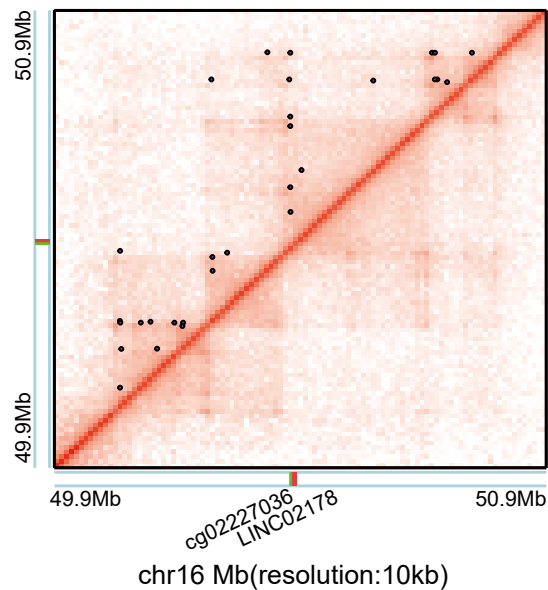**C****MCF-7**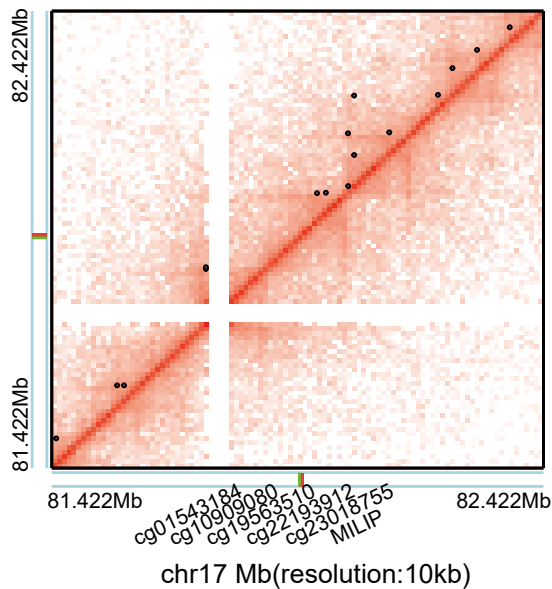**D****HMEC**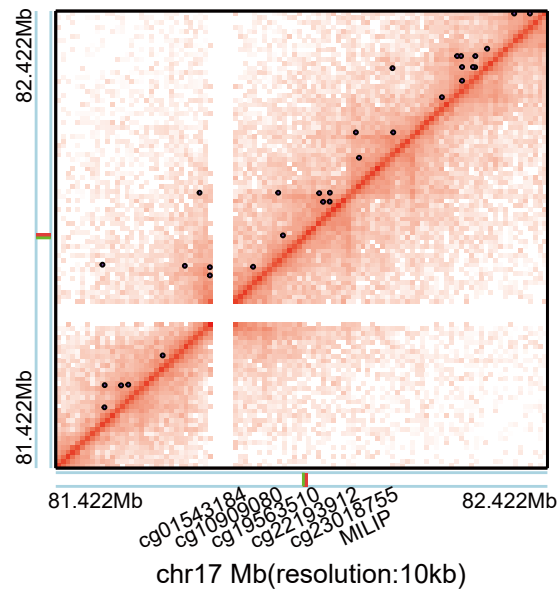

Supplement: Supplementary file 1 [file ijms-25-11048-s001.zip › Figure S3.pdf]

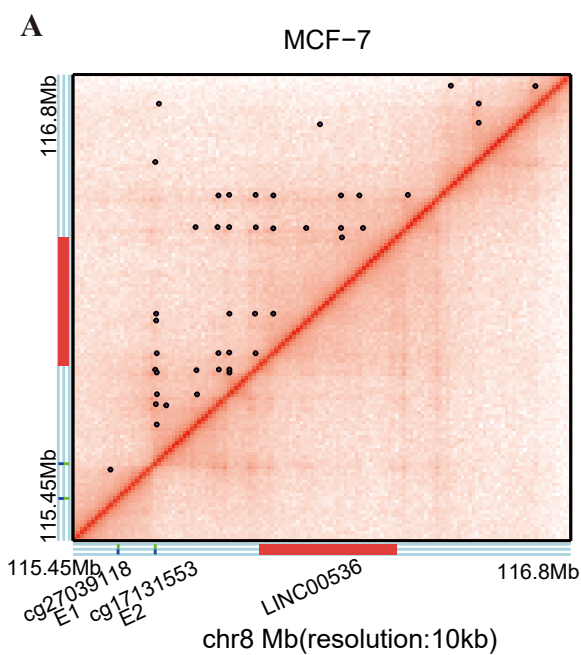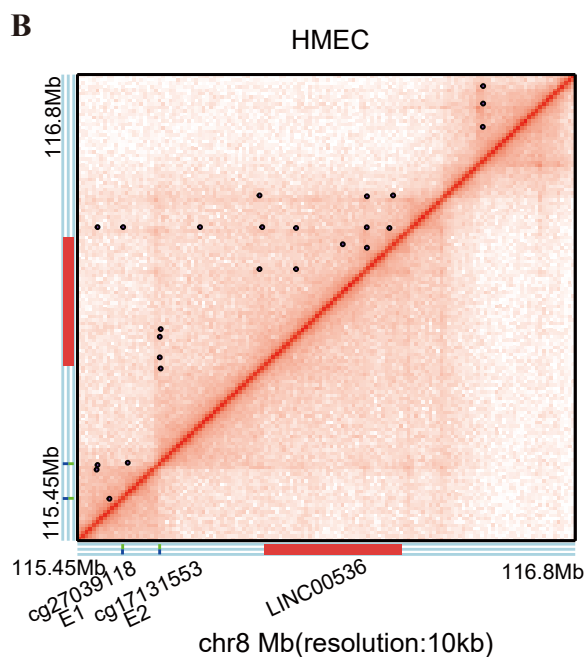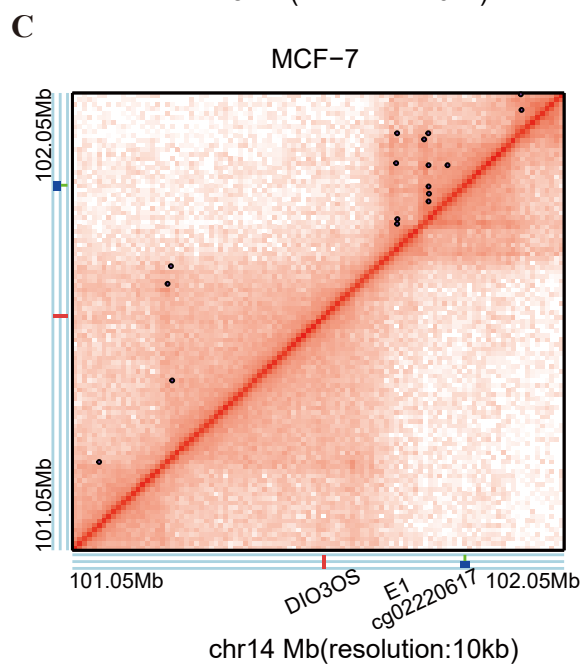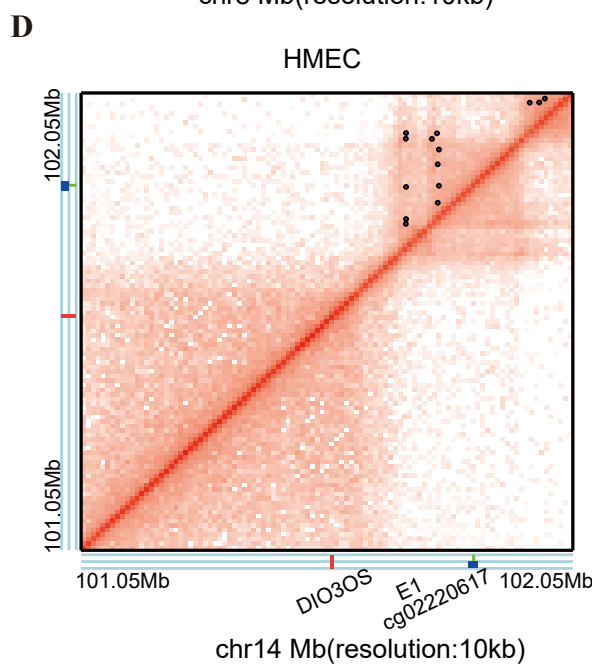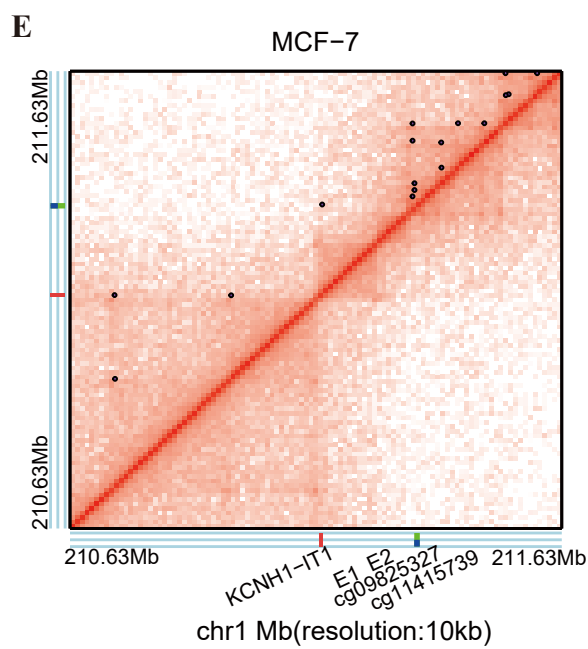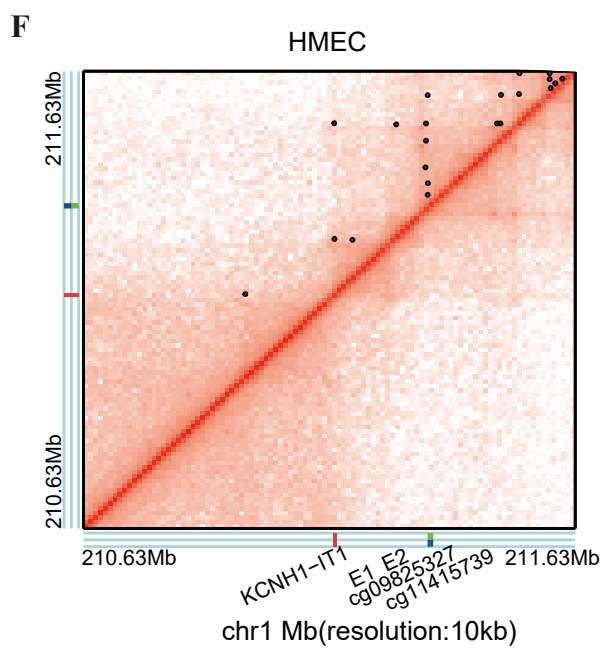

Supplement: Supplementary file 1 [file ijms-25-11048-s001.zip › Figure S4.pdf]
