# Supplementary material for: Effects of Differentially Methylated CpG Sites in Enhancer and Promoter Regions on the Chromatin Structures of Target LncRNAs in Breast Cancer
Source: Int J Mol Sci. 2024 Oct 15;25(20):11048. doi: 10.3390/ijms252011048 (PMC11507307; doi:10.3390/ijms252011048)
Supplement: Supplementary file 1 [file Figure S1.pdf › Figure S1.pdf]

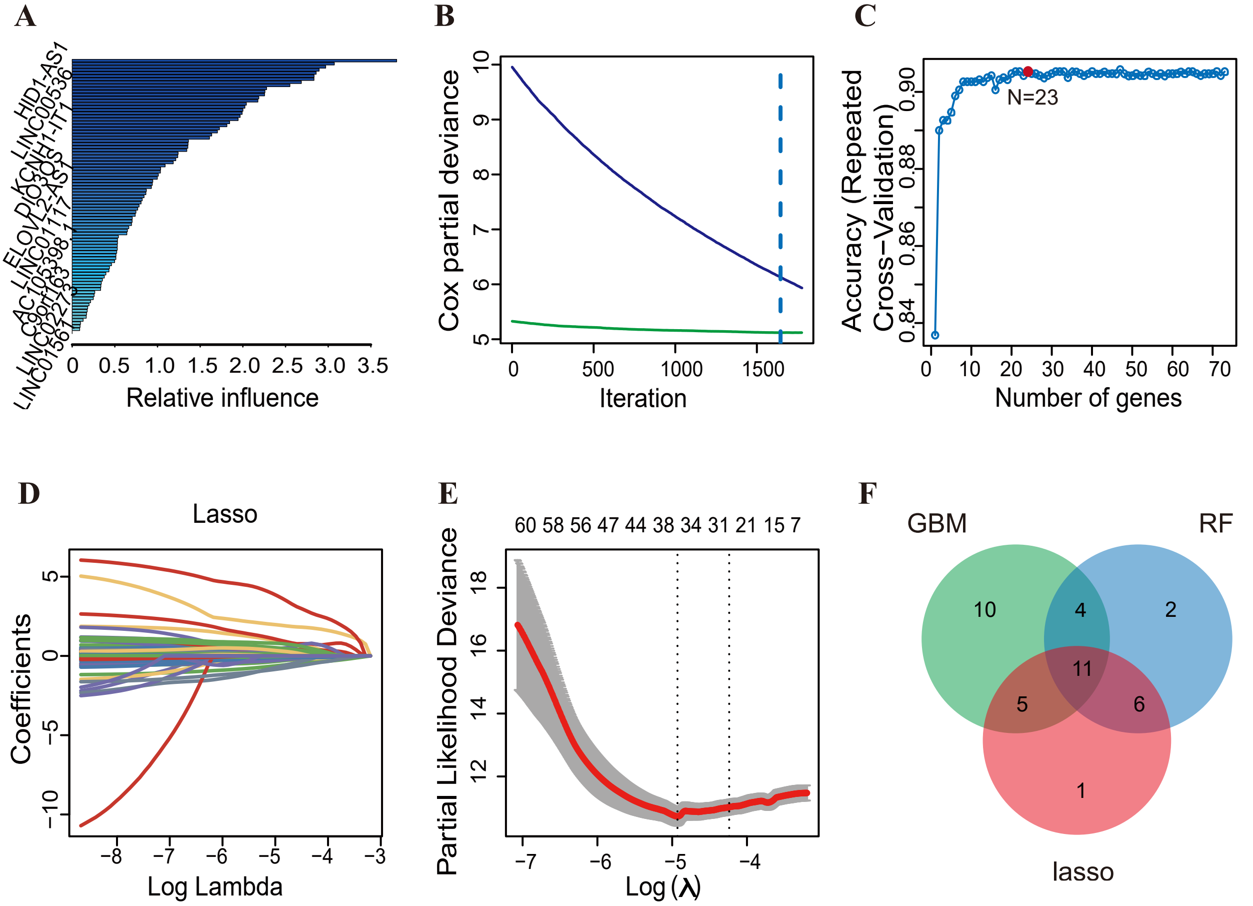
A
HID1-AS1
LINC00536
KCNH1-IT1
DIO3OS
ELOVL2-AS1
LINC01117
AC105398.1
C9orf163
LINC02273
LINC01561
0 0.5 1.0 1.5 2.0 2.5 3.0 3.5
Relative influence
B
Cox partial deviance
5 6 7 8 9 10
0 500 1000 1500
Iteration
C
Accuracy (Repeated Cross−Validation)
0.84 0.86 0.88 0.90
N=23
0 10 20 30 40 50 60 70
Number of genes
D
Lasso
Coefficients
−10 −5 0 5
−8 −7 −6 −5 −4 −3
Log Lambda
E
60 58 56 47 44 38 34 31 21 15 7
Partial Likelihood Deviance
12 14 16 18
−7 −6 −5 −4
Log (λ)
F
GBM
RF
10
4
2
11
5
6
1
lasso
